# Effect of external load on scapular upward rotation during arm elevation: the knot concept

**DOI:** 10.1186/s40634-016-0044-6

**Published:** 2016-02-03

**Authors:** Kazuya Madokoro, Masafumi Gotoh, Yoshihiro Kai, Tatsuyuki Kakuma, Takashi Nagamatsu, Naoto Shiba

**Affiliations:** Kurume University School of Medicine Graduate School, Asahi-machi, Kurume, Fukuoka Japan; Department of Orthopaedic Surgery, Kurume University Medical Centre, 155-1 Kokubu-machi, Kurume, Fukuoka 839-0863 Japan; Faculty of Health Science, Kyoto Tachibana University, Yamada-cho Oyake, Yamashina-ku, Kyoto Japan; Biostatistics Centre, Kurume University, Asahi-machi, Kurume, Fukuoka Japan; Department of Physical Therapy, Fukuoka Rehabilitation College, Hakataekimae, Hakata-ku, Fukuoka Japan; Department of Orthopaedic Surgery, Kurume University, Asahi-machi, Kurume, Fukuoka Japan

**Keywords:** Scapular upward rotation, Kinesiological change point, Knot, External load, Three-dimensional motion analysis

## Abstract

**Background:**

Failure of the scapulohumeral rhythm (SHR) is observed in patients with shoulder joint dysfunction. The SHR reportedly has a 2:1 ratio during scapular upward rotation with arm elevation. However, three-dimensional scapular motion analysis has indicated variations in this ratio according to the arm elevation angle. We observed 2 distinct patterns: the scapular upward rotation decreased after knot formation (type I) or increased after knot formation (type II) during arm elevation. In the present study, we aimed to identify the knot and investigate the influence of varying external loads on this kinesiological change point.

**Methods:**

We evaluated 35 healthy adult men (35 dominant-side shoulders) with a mean age of 20 ± 1.7 years (mean height: 172 ± 6.4 cm, mean weight: 65.7 ± 5.8 kg). Participants performed scapular plane elevation with no load or with an external load (1–5 kg) while sitting on a chair. The measured scapular upward rotation values were interpolated using the spline function and fitted to line graphs, and the change in these values was compared for various loads.

**Results:**

The estimated knot angles (standard error) in the no load condition, and with external loads of 1, 2, 3, 4, and 5 kg were 83.5 (2.9°), 81.2 (2.9°), 81.0 (2.9°), 76.1 (2.9°), 73.4 (3.1°), and 75.8 (3.1°), respectively. No significant difference was noted in the knot position at 1–2 kg (vs. unloaded), although the knot was significantly lower at 3–5 kg (3 kg: *p* = 0.01, 4 kg: *p* = 0.001, and 5 kg: *p* = 0.02). Moreover, we observed that participants either exhibited increased or decreased upward rotational momentum after knot formation.

**Conclusion:**

Our results confirm that the kinesiological change point (the knot) during scapular upward rotation occurred at lower angles in cases of increasing external loads.

## Background

**Fig. 1:**
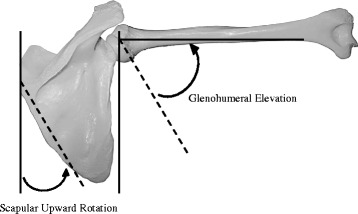
The scapulohumeral rhythm was determined by calculating the ratio of glenohumeral elevation and scapular upward rotation

Glenohumeral joint movement during arm elevation is coordinated by cooperation among the muscles around the shoulder joint (Kibler et al. 2012). However, failure of the scapulohumeral rhythm (SHR) is observed in patients with shoulder joint dysfunction (Ludewig and Cook 2000; McClure et al. 2006; Mell et al. 2005). Codman (1934) was the first to report on the SHR (Fig. 1), and many researchers have subsequently studied this topic. In particular, Inman et al. (1944) measured scapular upward rotation with arm elevation using the goniometric method, and observed that SHR consistently has a 2:1 ratio. Electromagnetic tracking devices and fluoroscopy have also been used for three-dimensional scapular motion analysis (Fayad et al. 2006; Giphart et al. 2013; Kon et al. 2008; Ludewig et al. 2009; McClure et al. 2001), and variations in the ratio according to the arm elevation angle have been reported (0.6–5.9:1 in Braman et al. 2009; 1.3–2.4:1 in Dayanidhi et al. 2005; 2.0–7.8:1 in Forte et al. 2009; 1.2–2.7:1 in Habechian et al. 2014; and 1.9–7.9:1 in McQuade et al. 1998).

The changes in the scapular upward rotation according to the arm elevation angle suggest the presence of a kinesiological change point or “knot” during this movement.

Various studies have reported that scapular upward rotation is influenced by external loads (Forte et al. 2009; Kon et al. 2008; McQuade et al. 1998). In particular, studies comparing loaded and unloaded scapular movement during arm elevation have reported that the scapular upward rotation decreased (Kon et al. 2008; McQuade et al. 1998), increased (Forte et al. 2009), or was not influenced (de Castro et al. 2014; Pascoal et al. 2000) by the external load. Hence, there is no consensus regarding the effects of external load on scapular upward rotation. However, previous studies have only compared static conditions at specific arm elevation angles, and to our knowledge, no studies have evaluated continuous scapular movement. We hypothesized that the knot can be identified during scapular upward rotation by interpolating discrete data, using the spline function defined in Equation (1), and subsequently observing the arm elevation angle and scapular upward rotation as continuous movements. Observation of the scapular rotation with external loads before and after knot formation during arm elevation would be useful in training in healthy subjects. Therefore, in the present study, we aimed to identify this knot during continuous scapular upward rotation, and the influence of external loads on this kinesiological change point.

## Methods

### Participants

We evaluated 35 healthy adult men (35 dominant-side shoulders) without any medical history of shoulder trauma or disorders (mean age: 20.7 ± 1.7 years, mean height: 172 ± 6.4 cm, and mean weight: 65.7 ± 5.8 kg). This study was approved by the Ethics Committee of Kurume University (no.: 09078), and all participants provided written informed consent.

### Measurement procedure

While sitting on a chair, participants performed scapular plane elevation with their dominant arm, from the relaxed downward position to the maximum elevated position at a constant speed over a 3-s period. Before measurement, the subjects practiced arm elevation without loading until they completed the motion in approximately 3 s. After an interval of 15 min, the subjects performed the experiments. This motion was performed in an unloaded condition or in a loaded condition with dumbbells that weighed 1–5 kg (1-kg increments). After sufficient practice was ensured, measurements were performed twice, and movements of the trunk, scapula, and humerus were monitored using an electromagnetic tracking system (Liberty; Polhemus, Colchester, VT) and analysis software (Motion Monitor®, version 8.43; Innovative Sports Training Inc., Chicago, IL). Among the measured values, we only included the data examined at 20–130° during arm elevation in the scapular plane.

**Fig. 2:**
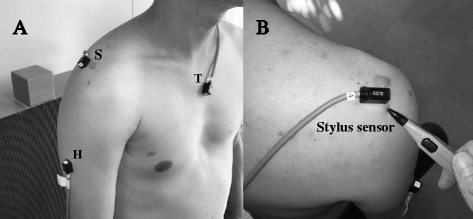
Magnetic sensors were fixed to the sternum, scapula (acromion), and humerus (in the neutral position) using double-sided tape on the participants’ dominant side. The bony landmarks of the thorax, humerus, and scapular were palpated and digitized with the stylus sensor (digitizer) to establish the anatomically based local coordinate systems. (**a**) S : Scapular sensor, T : Thorax sensor, H : Humerus sensor (**b**) The digitizing of by stylus sensor

The tracking system consisted of a transmitter, 7 sensors (receivers), stylus sensor (digitizer), and system unit. The transmitter generated a low-frequency electromagnetic field, and the sensors detected this field at a sampling rate of 120 Hz. The transmitter was placed on a wooden frame, and a global coordinate system was established by collating the major planes of the body to the appropriate positions. Three magnetic sensors were affixed to the sternum, scapula (acromion), and humerus (in the neutral position) using double-sided tape on the participants’ dominant side (Fig. 2-a-). Thereafter, 9 bony landmarks of the thorax, humerus, and scapular were palpated and digitized with the stylus sensor (digitizer) to establish the anatomically based local coordinate systems (Fig. 2-b). The sensors and the landmarks were placed in the required anatomic positions, according to the International Society of Biomechanics shoulder protocol (Wu et al. 2005).

Participants sat on a chair and relaxed their arm, and the cervical vertebra, humerus, and scapula were palpated and digitized for use as anatomical landmarks. Chest landmarks were set at the C7 spinous process (C7), T8 spinous process (T8), incisura jugularis (IJ), and xiphoid process (PX); humeral landmarks were set at the rotation center of the glenohumeral joint (estimated using the rotation method) (Biryukova et al. 2000, Veeger 2000), lateral epicondyle (EL), and medial epicondyle (EM); and scapular landmarks were set at the acromial angle (AA), trigonum spinae (TS), and inferior angle (AI). The longitudinal axis (Yt-axis) of the chest was drawn from the halfway point between the T8 and PX to the halfway point between the C7 and IJ; the lateral axis (Zt-axis) was perpendicular to the plane that was defined by the halfway points between the IJ and C7 and between the PX and T8 (pointing to the right); and the forward axis (Xt-axis) was defined by the intersection of the Yt- and Zt-axes. The humeral longitudinal axis (Yh-axis) was drawn from the halfway point between the EL and EM to the rotation center of the glenohumeral joint; the forward axis (Xh-axis) was perpendicular to the plane that was formed by the rotation center of the glenohumeral joint, EL, and EM; and the lateral axis (Zh-axis) was defined by the intersection of the Xh- and Yh-axes. The scapular lateral axis (Zs-axis) represented the AA and was drawn from the TS to AA; the forward axis (Xs-axis) was perpendicular to the plane that was formed by the TS, AA, and AI; and the longitudinal axis (Ys-axis) was defined by the intersection of the Xs- and Zs-axes.

### Identifying the knot of scapular upward rotation

The measured values of scapular upward rotation were interpolated using the spline function, and scapular movements were analyzed using a line graph to calculate the kinesiological change point during scapular upward rotation. The knot was defined as a point at which the scapular movement kinesiologically changed. The relationship between the arm elevation angle and the scapular upward rotation angle was presented using a two-piece linear regression model with one knot:

1 $$ \mathrm{Y} = {\upbeta}_0+{\upbeta}_1\mathrm{X} + \uplambda {\left(\mathrm{X}\kern0.5em -\kern0.5em \mathrm{c}\right)}_{+}+\upvarepsilon $$

where Y represents scapular upward rotation, X represents humeral elevation, and (X - c)_+_ is the spline function defined as max(0, X - c). Parameter β_1_ represents the slope for X ≤ c, while λ indicates the change in the slope when X > c. To estimate the model parameters for equation 1, it was necessary to fix the kinesiological change point (c). Hence, we estimated c by randomly setting 500 points between arm elevation angles of 20° and 130° as candidate knots. Using line graphs from these 500 points, we selected the model with the best fit to the original data (i.e., the minimum Akaike’s information criterion), and the kinesiological change point was considered to represent the knot. Duplicate measurements were performed for each participant (unloaded condition and loaded condition with 1–5 kg), and the knot was estimated for each condition. To investigate the reproducibility of the measured knot value, we estimated the intraclass correlation coefficient (ICC1,1).

In some cases, the measured knot value was markedly different between the first and second measurements. Thus, the absolute value of the difference between the 2 estimated values was determined, and was divided by the mean of the 2 values. The estimated values with a difference of ≥0.5 were extracted, and one of the 2 values with a large deviation from the median of all values was excluded as an outlier. Subsequently, we adjusted the estimated knot values with trial factors by using the linear mixed model (Twisk 2003), to determine the minimum mean square of the knot:

2 $$ {\mathrm{Y}}_{\mathrm{i}\mathrm{jk}}=\upmu +{\upalpha}_{\mathrm{j}}+{\upbeta}_{\mathrm{k}} + {\mathrm{b}}_{\mathrm{i}}+{\upvarepsilon}_{\mathrm{i}\mathrm{jk}} $$

Y_ijk_ represents the kinesiological change point value of the participant (i, in j repetitions with a load of k), μ represents the mean of the overall load, α_j_ represents the influence of repetition, β_k_ represents the effect of the load amount, b_i_ represents the individual effect for the participant (i), and ε_ijk_ represents the error.

To evaluate the influence of different external loads on scapular upward rotation, we compared the minimum mean square of the knot between the unloaded and loaded conditions (1–5 kg).

### Statistical analysis

All statistical analyses were performed using R software (version 3.1.0; R Foundation for Statistical Computing, Vienna, Austria), JMP® software (version 11; SAS Institute Inc., Cary, NC), and SPSS software (version 19.0; IBM, Tokyo, Japan). To evaluate the influence of external loads on scapular upward rotation, the rotation angle and SHR for every 5° of arm elevation were compared using two-way analysis of variance. In order to examine the effect of loads on the estimated knot value, a two-way mixed effects model was used, and the knot estimates for each loaded condition were compared with the unloaded condition using the Bonferroni adjustment for multiple comparison. The level of significance was set to 0.05 in all data analyses. Values are presented as mean values with standard deviation, except for the knot angle, which is presented as least mean square values with standard error.

## Results

### Scapular upward rotation angle

**Fig. 3:**
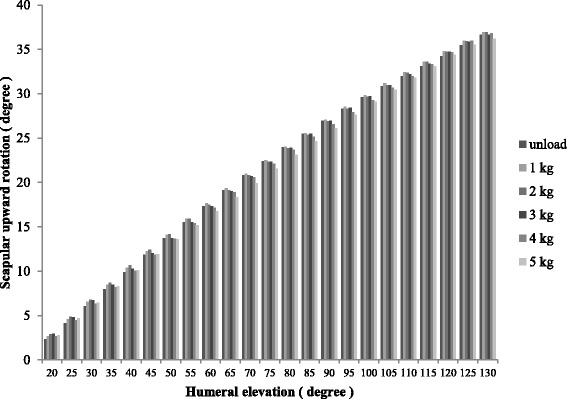
Scapular upward rotation and arm elevation for each external load (unloaded condition to loaded condition with 5 kg)

The mean scapular upward rotation angle (± standard deviation) for unloaded arm elevation was 36.6 ± 6.7°, compared to values of 37.0 ± 7.0°, 36.9 ± 7.1°, 36.7 ± 7.4°, 36.8 ± 7.3°, and 36.2 ± 7.2° for arm elevation with loads of 1, 2, 3, 4, and 5 kg, respectively. There was no interaction between the scapular upward rotation angle and the amount of external load, and no main effect was observed for the amount of external load on scapular upward rotation (df = 6.127, F = 1.218, *p* = 0.298) (Fig. 3).

### SHR

The mean unloaded SHR (± standard deviation) was 2.1 ± 0.6, compared to values of 1.9 ± 0.1, 1.9 ± 0.1, 1.9 ± 0.1, 1.8 ± 0.1, and 1.8 ± 0.1 with loads of 1, 2, 3, 4, and 5 kg, respectively. There was no interaction between SHR and the amount of external load, and no main effect was observed for the amount of external load on SHR (df = 1.428, F = 2.367, *p* = 0.12).

### Knot angle

The estimated unloaded knot position (± standard error) was 83.5 ± 2.9°, compared to values of 81.2 ± 2.9°, 81.0 ± 2.9°, 76.1 ± 2.9°, 73.4 ± 3.1°, and 75.8 ± 3.1° with loads of 1, 2, 3, 4, and 5 kg, respectively. No significant difference was observed in the knot position between the unloaded condition and loads of 1 kg (*p* = 0.33) and 2 kg (*p* = 0.29). In contrast, compared to the angle in the unloaded condition, the knot angle decreased significantly for all loads of ≥3 kg (3 kg: *p* = 0.01, 4 kg: *p* = 0.001, and 5 kg: *p* = 0.02) (Table 1).

**Table 1:** The estimated knot value and standard error of the knot for each external load

Loads	Least mean square	Standard error	*p*-value
Unloaded	83.5	2.9	Reference
1 kg	81.2	2.9	0.33
2 kg	81.0	2.9	0.29
3 kg	76.1	2.9	0.01^a^
4 kg	73.4	3.1	0.001^a^
5 kg	75.8	3.1	0.02^a^

^a^: Indicate significance after Bonferroni corrections

The knot was defined as a point at which the scapular movement kinesiologically changed

**Fig. 4:**
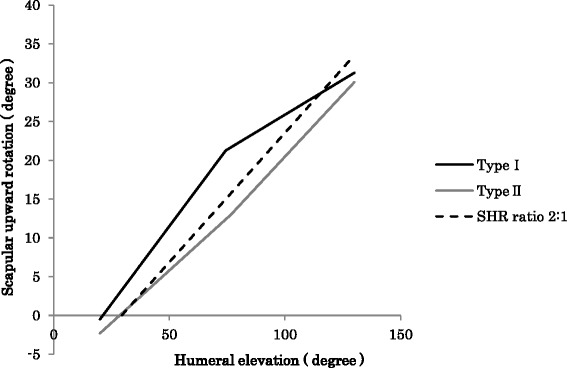
We observed 2 distinct patterns: the scapular upward rotation decreased after knot formation (type I) or increased after knot formation (type II) during arm elevation. The dashed line expresses the scapulohumeral rhythm ratio of 2:1 proposed by Inman et al. (1944)

With regard to scapular movements before and after knot formation, we observed 2 distinct patterns: the scapular upward rotation decreased after knot formation (type I) or increased after knot formation (type II) during arm elevation (Fig. 4). The frequencies of cases with types I and II were 83 and 17 % under the unloaded condition, 74 and 26 % with a 1-kg load, 77 and 23 % with a 2-kg load, 80 and 20 % with a 3-kg load, 74 and 26 % with a 4-kg load, and 74 and 26 % with a 5-kg load, respectively (Table 2).

**Table 2:** Frequency of the scapular upward rotation type after knot formation

	Type I	Type II
Unloaded	29 shoulders (83 %)	6 shoulders (17 %)
1 kg	26 shoulders (74 %)	9 shoulders (26 %)
2 kg	27 shoulders (77 %)	8 shoulders (23 %)
3 kg	28 shoulders (80 %)	7 shoulders (20 %)
4 kg	26 shoulders (74 %)	9 shoulders (26 %)
5 kg	26 shoulders (74 %)	9 shoulders (26 %)

Type I: The scapular upward rotation decreases after knot formation

Type II: The scapular upward rotation increases after knot formation

## Discussion

In previous studies, the influence of external loads on scapular movement during arm elevation was evaluated by setting the elevation angle at intervals of 20° (de Castro et al. 2014; Forte et al. 2009; Kon et al. 2008; McQuade et al. 1998; Pascoal et al. 2000). In contrast, we evaluated scapular upward rotation with arm elevation as a continuous movement, and were able to identify the knot by fitting the movement to a line graph using the spline function. Our findings confirmed that a knot was observed during scapular upward rotation with arm elevation, and increasing loads (≥3 kg) lowered the position of the knot as compared to the unloaded condition. However, loads of 1 kg or 2 kg did not significantly affect the knot position as compared to the unloaded condition.

When scapular upward rotation and SHR were compared according to external loads at 5° increments during arm elevation, we did not observe any significant differences in the scapular upward rotation angle or SHR, which confirms that external load does not influence these parameters. Similarly, Pascoal et al. (2000) analyzed scapular movement using 0–4 kg loads and an electromagnetic tracking device, and reported that external load did not influence SHR. Furthermore, de Castro et al. (2014) analyzed scapular movement during scapular plane elevation with a 5 % body weight load and videogrammetry, and reported that the amount of external load did not influence scapular movement. Thus, our findings are in agreement with previously reported findings.

Kon et al. (2008) reported that muscle control of the scapula is important to optimize the contractile force of the rotator cuff. Moreover, Forte et al. (2009) reported that the maximum load on the glenohumeral joint is achieved at 90° of elevation, and it is necessary to firmly compress the humeral head and glenoid fossa together via scapular upward rotation immediately before 90° of arm elevation to exert a sufficient elevation force. The knot appeared between the angles of 70° and 85°, shortly before the point of maximum moment from gravity was reached. As the scapula has rotated roughly 30° in this position, gravity induces a considerable amount of compression force across the shoulder joint. At arm elevation of 90°, the moment from gravity starts to decrease, and hence, a knot may be expected to form in this area. If there is no angular velocity, the net downward moment decreases in cases with negative acceleration. Moreover, if there is no negative acceleration, a greater amount of force would be needed to balance the increased moment due to gravity. Furthermore, the knot appeared at lower angles when loads of ≥3 kg were used (compared to the unloaded condition), which indicates that the scapula responded to the loads <3 kg. Nevertheless, the different kinematics of scapular upward rotation during arm elevation should be considered when training healthy individuals with this movement.

When we compared the slopes before and after knot formation, we identified 2 distinct patterns in which the scapular upward rotation increased or decreased after knot formation. In this context, the arm is elevated through coordination between glenohumeral joint elevation and scapular upward rotation via the muscles around the scapula (Kibler et al. 2012). Therefore, when one of the movements was stronger, the strength of the other increased to a greater extent after knot formation, indicating a complementary movement. However, the reasons for these dynamics are unclear, and further investigation is necessary to elucidate these reasons.

When the goniometric method was used to analyze scapular movement during arm elevation, humeral movement was considered dominant at low elevation angles and scapular movement was considered dominant at high elevation angles (Kibler 2006). However, when we examined arm elevation before and after knot formation, the change in the scapular upward rotation decreased in the latter segment for many subjects. This finding contradicts previously reported findings, and hence, further investigation is necessary to evaluate the interplay between these movements.

There are several important limitations in this study. First, we only used a three-dimensional motion analysis system, and did not perform any electromyographic analysis. Scapular movement is influenced by the force of muscles around the shoulder joint (Ebaugh et al. 2006), and the lower rotary couple (the inferior parts of the trapezius and serratus anterior muscles) plays an important role during scapular upward rotation (Inman et al. 1944). Thus, it is possible that the muscles around the shoulder joint may control the change in the knot and the subsequent change in momentum during scapular upward rotation in response to different amounts of external load. Second, in the present study, we measured the scapular upward rotation angle in the scapular plane, without observing its “rocking” and “tracking” on the rib cage. Anatomically, the scapular blade is separated from the rib cage by the serratus anterior and subscapularis muscles, and hence, the scapula can “rock” about any appropriate axis without any translation. Furthermore, the rib cage is curved in both the vertical and horizontal directions. We did not examine the influence of the upper and lower trapezius/serratus anterior muscles on scapular upward rotation. Third, as the sensors were fixed to the skin, an increased measurement error is possible when the arm elevation is ≥120° (Karduna et al. 2001, Cereatti et al. 2015). Fourth, the determination of the center of rotation of the humeral head may be limited due to the various studies reported previous (DeAngelis et al. 2015; Biryukova et al. 2000; Veeger 2000). Fifth, the scapular rotational velocity was not always constant, which might have influenced location of the knot. Sixth, experiments such as the present study cannot completely exclude skin motion; therefore, we affixed the acromion sensor to an area where the bone protrusion was as palpable as possible, in order to minimize this motion at that site.

## Conclusion

We found that a kinesiological change point (the knot) developed during scapular upward rotation. We also found that external loads of ≥3 kg significantly lowered the position of the knot (lower abduction angles) as compared to the unloaded condition. Furthermore, we observed 2 distinct patterns (an increase and decrease) for the momentum of scapular upward rotation with arm elevation after knot formation. These findings indicate that the elevation pattern varies among different patients, and may be useful in the development of customized treatments and training.

### Ethical disclosure

The institutional review board of Kurume University approved the study protocol (no.: 09078), and all subjects provided written informed consent before participating in the study.

## Abbreviations

C7: spinous process
T8: T8 spinous process
IJ: incisura jugularis
Yt-axis: the longitudinal axis along the chest
Zt-axis: the lateral axis along the chest
Xt-axis: the forward axis along the chest
Yh-axis: humeral longitudinal axis
Xh-axis: humeral forward axis
Xs-axis: scapular forward axis
Ys-axis: scapular longitudinal axis
SHR: scapulohumeral rhythm
PX: xiphoid process
EL: lateral epicondyle
EM: medial epicondyle
AA: acromial angle
TS: trigonum spinae
AI: inferior angle
